# Multiprofessional family health residency as a setting for education and interprofessional practices

**DOI:** 10.1590/1518-8345.4484.3450

**Published:** 2021-06-28

**Authors:** Heloísa Pimenta Arruda Araújo, Lucas Cardoso dos Santos, Thiago da Silva Domingos, Rúbia Aguiar Alencar

**Affiliations:** 1 Universidade Estadual Paulista “Júlio de Mesquita Filho”, Departamento de Enfermagem, Botucatu, SP, Brazil.; 2 Sociedade Beneficente de Senhoras Hospital Sírio Libanês, Saúde Corporativa, São Paulo, SP, Brazil.; 3 Universidade Federal de São Paulo, Escola Paulista de Enfermagem, Departamento de Enfermagem Clínica e Cirúrgica, São Paulo, SP, Brazil.

**Keywords:** Interprofessional Relations, Primary Health Care, Family Health, Interdisciplinary Communication, Health Care Team, Interprofessional Education, Relações Interprofissionais, Atenção Primária à Saúde, Saúde da Família, Comunicação Interdisciplinar, Equipe de Saúde, Educação Interprofissional, Relaciones Interprofesionales, Atención Primaria de Salud, Salud Familiar, Comunicación Interdisciplinaria, Equipo de Salud, Educación Interprofesional

## Abstract

**Objective:**

to know the experiences lived during the residency by graduates of a Multiprofessional Residency Program in Family Health that could contribute to the development of Interprofessional Education and/or Collaborative Practice.

**Method:**

a qualitative study with residents who entered a Multiprofessional Residency Program of a Brazilian public university in 2017, a period in which the theme of interprofessionality was implemented in the activities of the residency. Data was collected using an electronic form built from the theoretical framework of interprofessional education. Content analysis was used to process the data.

**Results:**

nine residents participated, distributed among the professions of Physical Education, Nursing, Nutrition, Dentistry, Psychology and Social Work, five of whom were female and with a mean age of 28.4. Two categories emerged: *the Residency as a setting for learning from the other, and the Residency as a setting for understanding the role of the other*. Interprofessional education and practice provided opportunities for the development of collaborative skills, enhancing teamwork and interprofessional work.

**Conclusion:**

the multiprofessional logic was evidenced in the resident’s practice; and the gradual insertion of activities such as case discussions, shared services and inter-sectoral actions aligned with the theoretical-methodological framework of interprofessionality favored an approach to interprofessional work.

## Abstract (pt)

**Objetivo:**

conhecer as experiências vivenciadas durante a residência por egressos de um Programa de Residência Multiprofissional em Saúde da Família que puderam contribuir para o desenvolvimento da Educação Interprofissional e/ou Prática Colaborativa.

**Método:**

estudo qualitativo com residentes que ingressaram em 2017 em um Programa de Residência Multiprofissional de uma universidade pública brasileira, período em que a temática da interprofissionalidade foi implementada nas atividades da residência. Os dados foram coletados por meio de formulário eletrônico construído a partir do referencial teórico da educação interprofissional. A análise de conteúdo foi utilizada no processamento dos dados.

**Resultados:**

participaram nove residentes distribuídos entre as profissões de educação física, enfermagem, nutrição, odontologia, psicologia e serviço social, sendo cinco do sexo feminino e média etária de 28,4. Emergiram duas categorias: a *Residência como cenário para a aprender com o outro e a Residência como cenário para compreender o papel do outro*. Educação e prática interprofissionais oportunizaram o desenvolvimento de competências colaborativas potencializando o trabalho em equipe e interprofissional.

**Conclusão:**

a lógica multiprofissional foi evidenciada na prática do residente e a inserção gradual de atividades como discussões de caso, atendimentos compartilhados e ações intersetoriais alinhadas ao referencial teórico-metodológico da interprofissionalidade favoreceu uma aproximação ao trabalho interprofissional.

## Abstract (es)

**Objetivo:**

conocer las experiencias vividas durante la residencia por egresados de un Programa de Residencia Multiprofesional en Salud de la Familia que puedan contribuir al desarrollo de la Educación Interprofesional y/o Práctica Colaborativa.

**Método:**

estudio cualitativo con residentes que ingresaron a un Programa de Residencia Multiprofesional de una universidad pública brasileña en 2017, período en el que se implementó el tema de la interprofesionalidad en las actividades de la residencia. Los datos fueron recolectados utilizando un formulario electrónico elaborado a partir del marco teórico de la educación interprofesional. Se utilizó el análisis de contenido para procesar los datos.

**Resultados:**

participaron nueve residentes, distribuidos entre las profesiones de educación física, enfermería, nutrición, odontología, psicología y trabajo social, cinco eran del sexo femenino y la edad promedio fue de 28,4 años. Surgieron dos categorías: la Residencia como escenario para aprender del otro y la Residencia como escenario para comprender el papel del otro. La educación y la práctica interprofesionales brindaron oportunidades para el desarrollo de competencias colaborativas, mejorando el trabajo en equipo y el trabajo interprofesional.

**Conclusión:**

la lógica multiprofesional se vio reflejada en la práctica del residente y la inserción paulatina de actividades como discusiones de casos, servicios compartidos y acciones intersectoriales alineadas con el marco teórico-metodológico de la interprofesionalidad favorecieron un acercamiento al trabajo interprofesional.

## Introduction

The complexification of health needs constituted by the determinants of the health-disease process, by the epidemiological change accompanied by a demographic transition and by the recognition of the weaknesses of the current health care models imposes comprehensive care as a normative horizon. Therefore, the importance of the health professionals working in collaborative teams in the search for more competent and resolving services is highlighted^(^ 1 ^-^ 2 ^)^.

Professional training has been a field of intense discussion because it is taken as a central element in the transformation of health work. The educational and health systems in different countries work around Interprofessional Education (IPE) as an innovative pedagogical strategy that results in the achievement of Collaborative Interprofessional Practices (CIPs) and in the qualification of assistance^(^ 1 ^,^ 3 ^)^.

With the intention of enhancing the development of collaborative skills for teamwork, IPE seeks to develop integrated and interactive learning between two or more health professions, allowing for greater understanding of the specific roles of each professional. The CIPs comprise a complex and dynamic process, in which professionals with different backgrounds work in an integrated and interdependent manner in sharing their knowledge, skills and attitudes^(^ 4 ^-^ 5 ^)^.

In this articulated training and assistance model, the CIPs intend to improve the quality of health care that occurs from effective teamwork, which in turn is based on the development of IPE^(^ 1 ^,^ 4 ^-^ 5). It is argued in favor of the effectiveness of acting in an interprofessional team centered on the patient when compared to the work of the health professional carried out in isolation^(^ 6 ^)^.

The World Health Organization (WHO) sparked an important movement from 2010 onwards by launching the Framework for Action in Interprofessional Education and Collaborative Practice^(^ 7 ^)^. And the Pan American Health Organization (PAHO) influenced the process of implementing IPE and highlighted the need to think about initiatives aimed at ensuring the sustainability of the topic in the academic environment^(^ 8 ^-^ 10 ^)^.

In the Brazilian context, some initiatives and political advances stimulated changes in vocational training that converged with the proposal of Interprofessional Education and Collaborative Practice (IECP), among which are the implementation of the Unified Health System (*Sistema Único de Saúde*, SUS) in 1990, the Family Health Program in 1994, the National Curriculum Guidelines in 2001, and Resolution No. 569 of December 8^th^, 2017. Among the common assumptions, principles and guidelines for undergraduate courses in health, this resolution includes interprofessional work, which must be expressed in the training of a professional able to work for comprehensive health care^(^ 11 ^)^.

The incentives of the Ministry of Education (MoE) and the Ministry of Health (MoH) drove the reorientation of the curricula of health courses, both at the undergraduate and graduate levels, through some programs: Incentive Program for Curricular Changes in Medical Courses (Promed), in 2002; Multiprofessional Residencies in Health, in 2005; National Program for the Reorientation of Professional Training in Health (Pró-Saúde), in 2006; and Education through Work-Health Program (PET-Saúde) in its different modalities, starting in 2008, and which currently has PET-Saúde Interprofessionality^(^ 12 ^-^ 13 ^)^.

Multiprofessional Health Residencies, created from the enactment of Law No. 11,129 of 2005, represent a possibility to contribute to the reorientation of the care model through professional training by requiring learning with, about and for the other, in addition to acquisition of skills that can support the planning of shared actions and patient-centered care, placing them as the main actors in this process^(^ 14 ^)^.

Despite the already recognized contributions of the IECP to the training of health professionals, national and international studies reinforce the need to incorporate the theoretical framework of interprofessionality in the training of residents^(^ 2 ^,^ 14 ^-^ 15). In a complementary way, the development of scientific research studies favors and subsidizes the construction of evidence about the implementation of the IECP in professional training. In this scope, the need to monitor and evaluate the training path of health professionals using the theoretical framework of the IECP is highlighted^(^ 16 ^)^.

Given the context presented, the development of this study is justified, which aims to explore an educational initiative using the theoretical framework of the IECP in the Multiprofessional Residency Program in Family Health (*Programa de Residência Multiprofissional em Saúde da Família*, PRMSF). Therefore, the objective was to know the experiences lived during the residency by graduates of a Multiprofessional Residency Program in Family Health that could contribute to the development of Interprofessional Education and/or Collaborative Practice.

## Method

It is a qualitative research^(^ 17 ^)^, which focused on the practices of the IEPC and the value attributed to them by the graduate students from the PRMSF, taking into account the steps recommended by the Consolidated Criteria for Reporting Qualitative Research (COREQ)^(^ 18 ^)^.

The research was carried out in a public higher education institution, located in Botucatu-SP, Brazil. At the time of this study, the municipality had 144,820 inhabitants, of which 40.5% were covered by 17 Basic Health Units in the Family Health model and 57.0% were covered by eight Basic Health Units (BHUs) in the traditional model, and two School Health Centers (SHCs)^(^ 19 ^)^.

In 2003, the PRMSF was offered for the first time at the institution analyzed only for medical and nursing courses. As of 2008, 10 vacancies were offered, distributed in the professional categories as follows: one vacancy for the courses of Physical Education, Physiotherapy, Nutrition, Dentistry, Psychology, Social Work, and four vacancies for the Nursing course, having 59 graduates to the present day.

The theoretical activities of the PRMSF are distributed in a module common to all professions and specific, totaling 1,152 hours, developed within the scope of the university, with the objective of developing the theoretical foundation of health work in the SUS and in the reflection of the practice.

Among the teaching strategies used there is resolution of cases through Problem-Based Learning (PBL) with the participation of residents from two years of training and from different professional categories, with the facilitation of tutors and coordination of the residency. The meeting between the residents and their tutors in the same professional category takes place in the tutoring space in order to develop specific discussions in the professional area, in addition to enabling the meeting to reflect on the portfolio, built from the practice experienced by the residents and in the form of narratives. Finally, theoretical classes are offered that take place with other residency programs of the referred institution in order to develop scientific and epidemiological reasoning, which are common to the professional categories.

Practical activities take place in the services of the Health Care and Psychosocial Network, such as Family Health Units (FHUs), School Health Center, Family Health Support Center (*Núcleo de Apoio à Saúde da Família*, NASF), State Schools, Emergency Department, Psychosocial Care Center and Epidemiological Surveillance Center, serving 4,608 hours, with a total of 5,070 hours over the two years. The choice of the practice scenarios is made by the coordination of the residency, preceptors, tutors and municipal management.

Each PRMSF class is divided and distributed into four FHUs, each team consisting of three or four residents who remain in the same unit during the two years of the residency. The residents from Physical Education, Physiotherapy, Nutrition, Psychology and Social Work have a progressive insertion in the activities developed by the NASF team in the municipality, with emphasis on matrix support and inter-sectoral meetings. Nurses and dentists, on the other hand, maintain their insertion in the activities of the FHUs.

In this context, in 2017 the PRMSF started to discuss the theoretical background of the IECP^(^ 1 ^-^ 2 ^,^ 5 ^,^ 8,12) in theoretical and theoretical-practical activities, in the tutoring meetings, and in the case discussions seeking an approximation to the development of interprofessional work.

The inclusion criteria of the participants were as follows: having an interest in collaborating with this research, having obtained the title of specialist by the program in February 2019, and joining the program in March 2017. The choice to include only this group of graduates occurred because the discussion of the IECP started intentionally and was implemented in the activities of the PRMSF in 2017, a theme that was not yet addressed at the time by the other programs. No exclusion criteria were outlined.

To select this sample, all graduates who obtained the title of Specialist in Family Health in February 2019 were invited to participate; however, there was a single refusal justified by lack of time to answer the form.

Data collection was conducted between the months of June and July 2019 by a female researcher, graduated in Nursing and, in that period, a resident of the second year of the aforementioned Residency Program. The fact of being a resident inserted in the program represented a strategic methodological element, whose objective was to establish an identification between participant and researcher, considering the previously established relationship, with a view to achieving greater adherence of the participants to the research.

It is important to highlight that the rigors of the theoretical-methodological point of view were followed to carry out a research study with a qualitative approach. In this study, the team’s expertise mattered for the elaboration of the instrument and data analysis.

After the telephone contact to inform the objective of the research and for clarification of doubts, the researcher shared the data collection instrument through a research management application (Google Forms).

The use of the data collection strategy in an online environment was considered thinking about the benefits that its use brings, such as the possibility for the participant to access the research at any desired time in cases of asynchronous strategies, for the researcher to follow the development of the research as the data is being filled in the digital platform, and for the non-involvement of the researcher with the participants, providing neutrality^(^ 20 ^)^.

Based on the framework of IPE^(^ 1 ^,^ 6 ^-^ 8 ^,^ 10 ^,^ 12), after careful elaboration and planning, the online data collection instrument was initially implemented in a pilot study with the objective of improving it and generating in the researchers reflections about the data found and, with that, the achievement of the proposed objective. The results found in this stage were not used in the research, but allowed adjustments to the instrument and greater ability and preparation of the researcher for the data collection process.

When declaring to be in agreement with the Free and Informed Consent Form (FICF), the participant was directed to the first part of the form, which contained closed questions for demographic, occupational (name, gender, age and professional category) and academic (educational institutions attended during their training) characterization.

In the second part, to explore the research theme, open questions were elaborated to be answered by the participants, with no character limit, in order to provide free space for reflection and description of their experiences and perceptions.

The questions were elaborated from publications with a qualitative approach and object of similar studies^(^ 1 ^-^ 2 ^,^ 5 ^,^ 8,12,14,21) that considered items to be explored in the composition of the questions such as those described in Figure 1, in which they deal with the following: the reason for the professional choice, the experiences during graduation and residency in relation to the IECP, and the professional categories that stood out in their teaching-learning process.

**Figure 1:** Items explored in the composition of the instrument's questions. Botucatu, SP, Brazil, 2019

Items explored in the composition of the questions	Questions
Motivation for the professional choice	Why did you choose your undergraduate course?
Experiences with other professional categories throughout graduation	During your graduation, did you have the opportunity to take classes/activities with students from courses in other areas? If so, tell us how these classes/activities took place:
Interaction with professional categories, with the possibility of being another member of the residency or the health service worker in question	Which profession(s) would you list as most important for your teaching-learning process during the residency? In your answer, consider the professions of the health services team and of the residency.
Learning with, about and for each other about attitudes, perceptions, roles, responsibilities and relationships with health care.	From the profession(s) listed in the previous question, talk about what you learned about attitudes, perceptions, roles, responsibilities and relationships with health care?
Experiences with a professional, another resident and/or patient, contextualizing its importance for your training.	Write a text in the format of a reflective narrative about some experience that occurred during the residency in which you had the opportunity to perform/experience with some professional or other resident, contextualizing its importance for your professional training.

The third part of the form referred to the composition of a Reflective Narrative^(^ 22 ^)^ in which the participants should narrate some experience that occurred during the residency with another professional, resident and/or patient that could be considered remarkable for their professional training.

It is worth mentioning that the decision to ask the participants to write a reflective narrative was made because it is already a teaching strategy used in the context of the PRMSF and also presents itself as relevant in teaching and research. This reflection on the action and the past moment allows for new meanings and greater understanding of what has been experienced^(^ 22 ^)^.

The time to answer the questions and build the narrative was free, lasting a mean of 20 minutes depending on the reflective narrative constructed by each of the participants.

To guarantee the participants’ confidentiality, an alphanumeric code composed of the letter “R” followed by a one-digit number was used when excerpts from the questions that comprised the first part of the instrument were used. In the case of excerpts extracted from the reflective narratives, the letter “N” was used, also followed by a number.

Data processing was guided by Content Analysis, while the phenomenon studied was interpreted in the light of the theoretical-conceptual framework of the IECP^(^ 1 ^-^ 2 ^,^ 5 ^,^ 8 ^,^ 12 ^)^.

Content Analysis is considered as a set of techniques with the use of systematic and objective steps to describe diverse discourses and highlight the nature and relative strengths of the stimuli to which the individual is subjected^(^ 23 ^)^.

The analysis of the material generated from the application of the instrument used followed three stages: floating reading, in which the first contact with the analyzed material took place; classification of the elements of the texts in a system of categories according to their meaning; treatment of the results obtained; and interpretation^(^ 23 ^)^.

Coding took place from the participants’ reports, which were cut and grouped thematically, giving rise to two central categories, as shown in Figure 2 below:

**Figure 2:** Presentation of the theoretical framework, and of the central and empirical categories and subcategories. Botucatu, SP, Brazil, 2019

Theoretical Framework	Central Categories	Empirical Categories	Subcategories	Representation of the Subcategories
Interprofessional Education	The Residency as a setting for learning with the other	Interpersonal relations	Resident-Resident	R1, R2, R3, R4, R5, R6, R7, R8, R9
			Resident-Health professional	R1, R2, R3, R4, R7
			Resident-Community	R2, R5, R6, R9
			Inter-sectoriality	R4, R7
	The Residency as a scenario to understand the role of the other	Enhancing the CIP*	Recognize the performance of other professional categories	R1, R2, R3, R4, R5, R7, R8, R9
			Deconstruct previous concepts	R3, R4
			Skills to be incorporated in the own practice	R1, R2, R3, R4, R5, R8, R9
			Recognize professional limits	R2, R3, R4, R5, R8

* CIP = Collaborative Interprofessional Practice

**Figure 3:** Characterization of the research participants. Botucatu, SP, Brazil, 2019

Participant	Gender	Age	Professional Category	HEI	Undergraduate IPE experience
R1	Female	26	Psychology	State public - In-person	Moments with psychologists, nurses, physicians, technicians during an internship in Primary Health Care services.
R2	Male	29	Odontology	State public - In-person	Did not have any contact with other professional categories. Mentions as a hindrance the physical structure of the institution that was isolated from other courses. Sought an extension project that provided interaction with Biology, Biomedicine, Nursing, Pharmacy, Physiotherapy, Medicine, Psychology and Veterinary Medicine.
R3	Male	25	Nutrition	State public - In-person	Did not have any contact with other professional categories. Sought an extension project and had contact with physicians.
R4	Female	32	Nursing	State public - In-person	A course in conjunction with medical students.
R5	Female	26	Nursing	Federal public - In-person	Several disciplines that provided contact with other professional categories in the health area.
R6	Female	25	Nursing	State public - In-person	A course in conjunction with medical students.
R7	Male	28	Social Service	Private - In-person	Did not have any contact with other professional categories. Sought an extension project and had contact with Psychology students.
R8	Female	25	Nursing	State public - In-person	A course in conjunction with medical students.
R9	Male	40	Physical Education	State public - In-person	Did not have any contact with other professional categories.

As explained, the FICF was offered electronically, before the presentation of the data collection instrument, with guidance on the objectives of the research and the non-mandatory nature of participation, according to Resolution No. 466/2012^(^ 24 ^)^. The present study was approved by the Research Ethics Committee on 06/07/2019, under opinion No. CAAE 13990519.0.0000.5411.

## Results

Figure 3 shows the main characteristics of the participants. There is majority of females, with a mean age of 28.4 years old, from undergraduate courses linked to public institutions and without carrying out theoretical and practical academic activities shared with students from different courses, known as a uni-professional training model.

The analysis of the qualitative corpus allowed constructing two categories: *The Residency as a scenario for learning with the other and The Residency as a scenario for understanding the role of the other*.

The *Residency as a scenario for learning with the other* category deals with the resident’s strand of learning with different actors represented in the participants’ speeches by service professionals, other residents and users.

The close relationship with service professionals was reported as an opportunity for sharing knowledge and planning care in a shared manner: *[...] discussions with professionals from the territory were essential, as they accumulated many experiences about the community, as well as the territory (N7)*; and [...] *the team’s presence was always very clear, extremely important for the learning process in a residency* (N1).

The meetings that allowed for the interaction of the different actors occurred at different times, as reported: [...] *there were shared consultations, team discussions of the cases we thought were more complex, multiprofessional home visits, inter-sectoral work* (N4). However, the performance of participants with different professions occurred mainly through case discussions considered as [...] *spaces in which we could see the convergence points between professional activities and we unified them in order to build a common care plan (N6), as well as an opportunity to discuss, think about strategies, identify situations that often went unnoticed by me* [...] (R2).

Shared services were mentioned as punctual and specific events based on the identification of the participant’s need to access the core knowledge of another profession, as explained in the following speech: *[...] enhanced my practice with information and techniques, but also made me able to recognize the limits of my profession and the importance of a joint approach* (R5).

The participants pointed out that the work with professionals from other services, carried out with different equipment from the Health Care Network, resulted in an approach to the community and its real social and health needs through planned collective activities, as highlighted in the report: *[...] activities were planned in conjunction with the health unit, social equipment and residents, which in addition to developing teamwork skills [...] political and critical awareness of the community context, allowing us to look beyond the health units and services* (N4).

In addition, the co-responsibility between professionals and residents and the users was significant when placing the latter at the center of care: *[...] this co-responsibility between professionals and the user is understood in the work routine and builds meaningful experiences* (R6).

The interaction with the service users was also perceived as an important moment for strengthening relationships and the perception and feeling of belonging to the service team by the resident: *[...] I did the whole approach, all the orientations, all the questions necessary to, thus, think [resident and user] in the next steps* (N2); and *[...] creation of important links with service users who started looking for us and trusting in our work* (N5).

The positive experiences from the close relationship with the community were mostly remembered through group activities that allowed for the articulation of technical and popular knowledge to meet the collective needs, not just individual ones.

The relationship between the participants themselves was rated as significant by all, being considered a unique opportunity to learn from each other’s experience and practice: *[...] my co-worker had other experiences, which contributed to a better understanding of the demand to be worked on, as well as other intervention instruments* (N7).

Some also pointed out which professional categories played a different role during their residency and the positive impact on their professional training focused on CIP, with the category most mentioned by participants being Psychology, as made explicit below: *[...] the possibility of exchanging experiences with the professional in question [Psychologist] helped me to recognize, in a more sensitive way, some attitudes of the patients, allowing approaches and orientations closer to reality* (R3).

It was also reported that learning from a colleague with a different professional background allowed for the acquisition of knowledge that enabled approaches and guidelines that were closer to the reality of the users, the family and the community: *[...] Social Assistance opened my mind to other ways of seeing and intervening in health [...]. Their attitudes always took into account the personal and social context of the individuals* (R9).

It was possible to verify that, through the participants’ interactions and relationships with the different actors involved in the assistance, the approach to the user was expanded even when performed individually, due to the establishment of connections between the different knowledge areas: *[...] I learned to have qualified listening and [to do so] embrace the suffering of the other, in addition to learning how to handle these issues within my limitations of the profession* (R8).

The Residency as a scenario to understand the role of the other category deals with the relevance of knowing the different roles of the professions as a way to enhance teamwork and collaborative work.

When analyzing the narratives, it was noticed that the practice scenarios of the residency made it possible to identify and understand the role of the other, as evidenced in the excerpt below, in which a participant starts to see different forms of performance of a colleague with professional training different from his: *[...] Nursing has surpassed the field of direct patient care, adding knowledge mainly in the managerial area* (R3).

In addition, it also presented itself as an opportunity to deconstruct previous concepts about other professions: *[...] I had the view of a [Physical Education professional] concerned only with aesthetic issues and sports performance* (R3); and [...] when I understand the role of each professional in the team, I broaden my view and the possibilities that can be offered (R4).

On the other hand, there were reports that portrayed a stereotyped and limited understanding of the professions: *[...] the professionals collected information about the patient’s entire life history. The physical educator [...] recommending several specific exercises, the nutritionist made nutritional adjustments [...]* (R3).

In line with this fragility, it was identified in some reports that the term sometimes used was “multiprofessional”, which evidenced lack of mastery of the IECP terms by the graduates.

Approaching different professional categories also made it possible to reflect on them, which enabled the participant to see in the other the skills and attitudes to be incorporated into their professional practice, as reported: *In nutrition, I realized that the professional’s attitude is [...] organized and [...] based on the evidence [...]. On the other hand, with the Social Worker, I could see [...] that their attitudes always took into account the personal and social context of the individuals (R9); and With dental professionals, I learned theoretical and technical issues, specific to the area, which provided me with a greater repertoire in my guidelines* (R8).

Consequently, the learning of service professionals and graduates about the role and importance of the other was considered positive for the care offered, as made explicit below: *I realize, then, that there are many gains when the professionals understand and believe in interprofessional work, both for professional improvement and for direct patient care* (N8).

A counterpoint to the recognition of the limits of the professions’ performance is what they have in common, making this encounter a powerful space for the construction and expansion of care. However, it is often not performed by the services, which give preference to activities performed in a multidisciplinary way with little interaction between those involved.

Considering an arduous path for the IECP, some factors were identified as barriers to its execution, as pointed out by the following participants: *[...] the way the services are organized, so that they don’t encourage or collaborate for them to take place* (N4); *[...] delineating the roles of each professional is something that brings a lot of tension. For some, interprofessional work is a threat to their autonomy as a professional (R6); and [...] an arduous path, as it involves different subjects from different backgrounds, but at the same time it’s effective, efficient and rewarding* (N3).

## Discussion

As evidenced in the results, the Multiprofessional Residency in Health, as it is organized in the logic of teamwork and network, presents itself as a type of interprofessional training by allowing for the articulation of different professional views that make up the programs and services, as well with the residents and professionals and those with other sectors, users and their families, when guided by the assumptions of the CIP^(^ 25 ^)^.

In Brazil, even though the SUS is based on the principles of universality, integrality and equality, and with a focus on teamwork, the training process remains organized in essentially uni-professional curriculum models, bringing as a consequence the fragmented and isolated work of the health professions^(^ 26 ^-^ 29 ^)^.

In contrast, international experiences such as in Canada and the United States are more advanced when reporting that the CIP has enabled integrated teamwork, presenting positive impacts on care in primary care services and bringing the following as the most prone professional categories to this practice: social workers, nurses, pharmacists, physicians, mental health professionals and nutritionists^(^ 2 ^)^.

In this sense, converging with the finding in the literature^(^ 2 ^)^, the participants of this study identified and recognized the resident psychologist as the one with an important role in the training of a colleague from another profession.

The transformations in the educational and health systems must occur at the levels of interpersonal relationships, processes of curricular changes and health and education policies, in an interdependent and articulated manner, providing for the reorganization of the health work process and the improvement of the assistance offered^(^ 1 ^,^ 5 ^)^.

Therefore, as stated by the research participants, the IPE in the perspective of learning with, about and for the other has been pointed out as being able to promote changes in professional training by improving teaching-service integration and professional relationships in order to seek tackling social and health problems^(^ 1 ^,^ 30 ^-^ 31).

In this context, as a WHO initiative, the Framework for Action in Interprofessional Education and Collaborative Practice reinforces the role of the health and education systems, mapping initiatives to the time, and being an excellent guide for national and international experiences^(^ 7 ^)^.

The Brazilian IPE experiences have involved students from different professional categories - social workers, physical educators, nurses, pharmacists, physiotherapists, speech therapists, physicians, nutritionists, psychologists and occupational therapists - with the use of pedagogical practices at the beginning of graduation made possible in the context of the health work. The students’ perspective of these experiences emphasizes the importance of the teacher as a mediator in the training process for critical and intentional reflection around the deconstruction of stereotypes, the sharing of practices and knowledge, and learning with and about the other^(^ 14 ^,^ 32 ^)^.

The teaching-service integration discussed in the Dutch scenario reiterates patient involvement with chronic conditions in the context of IPE. University students in Nursing, Physiotherapy, Speech Therapy and Medicine participate in three modules in which patients with chronic conditions share their personal experiences with regard to the care offered in the health services. This environment of interaction between undergraduate students and patients organized in small groups and facilitated by a teacher resulted in a more comprehensive and empathic approach, directing the students towards the practice of person-centered care^(^ 27 ^)^.

In this way, changes in the CIP perspective occur as health care focuses on the user, being recognized as a shared process that also integrates families, the community and different professionals^(^ 26 ^-^ 29 ^)^. Three dimensions allowed identifying an effective interprofessional integration: the students’ profile, the contact with the team and the duration, allowing for greater experience and greater knowledge in relation to the service^(^ 32 ^-^ 33 ^)^.

The experiences and the exchange of experiences with users in the different contexts and devices of the territory and in group activities, in the context of Primary Health Care, were mentioned by the participants as ideal opportunities for interprofessional training and direct contact with the needs of the population, being an intense learning process for students, service professionals and users^(^ 33 ^-^ 34 ^)^.

Therefore, the co-responsibility and the patient-centered care brought in the speeches must be understood as domains and essential elements for teamwork and interprofessional work^(^ 34 ^)^. By reinforcing the sharing of responsibilities between professionals and the user, the CIP shows itself as a powerful strategy in the recognition and reinforcement of practices that place the patient at the center of their care, as well as an initiative that builds more symmetrical relationships between the actors involved in this process^(^ 26 ^-^ 29 ^)^.

The experiences reported about the IPE point out important changes in the profile of the graduates of the Residency Program, analyzing evidence in the understanding that learning about the role of a colleague from another profession allowed them to qualify their work in health. In addition, it was understood that learning with and about the other results in overcoming the lack of knowledge of other professional groups, in training aimed at teamwork and in the development of skills necessary for collaboration^(^ 1 ^,^ 3 ^,^ 30 ^,^ 32 ^,^ 35).

Competences are considered the combination of knowledge, skills and attitudes, which can be divided into three types. Common competences are those similar across the professions, which can be practiced by all the professionals. Specific competences distinguish professions from one another, while collaborative competences configure the skills necessary for teamwork to be effective^(^ 36 ^)^.

Based on the participants’ recognition of the boundaries between professions, which are sometimes mobile and overlap, attention is paid to the importance of collaborative competences in the profile of the health professionals, as they allow for the resolution of interprofessional conflicts, shared decision-making, clarity of roles, attention centered on the user/family/community, and effective interprofessional communication^(^ 33 ^-^ 34 ^,^ 37).

The positive impact on the assessment of the effectiveness of CIP in patient care and in the work dynamics in the health services was demonstrated through a systematic review study, indicating the following as results: adherence to care protocols, user satisfaction with the care received, knowledge sharing in decision-making by the professionals, collaborative practice, and reduction of errors in the assistance offered^(^ 2 ^)^.

Such evidence is in line with the results of this research and other findings in the national^(^ 4 ^-^ 5 ^,^ 14) and international^(^ 2 ^,^ 27 ^-^ 28 ^,^ 30) literature, since the interpersonal and interprofessional relationships operated by the residency provide opportunities for reflection-action, different moments of exchange, better planning of care, understanding of the health and social demands of users and their families, as well as the acquisition of collaborative skills that enabled better guidance of the assistance provided.

When thinking about interprofessional work, it is important to consider that one of the conditions for its occurrence is that the team is multiprofessional. However, in a team organized according to the multiprofessional logic, there is not necessarily interaction and collaboration across the professions. On the other hand, an interprofessional team articulates based on the interactions of different professionals with a view to collaboration and around a common and explicit objective, which is caring for the user^(^ 1 ^,^ 4 ^-^ 5).

The literature shows some barriers to the non-realization of the IECP that converge to the participants’ speeches, such as the lack of understanding and little knowledge about the role of other professionals, in addition to the understanding of interprofessionality as a synonym of multiprofessionality^(^ 3 ^,^ 38 ^)^.

Furthermore, the training guided by the biomedical hegemony and the Flexnerian health model, competitiveness in the care field, fear of losing professional identity^(^ 1 ^,^ 30 ^,^ 39), the uni-professional curriculum structure that hinders common learning, the historical roles of each profession, and the lack of understanding of the social contract that each profession has with the patients^(^ 12 ^)^ also limit the adoption of these practices.

Considering these difficulties and barriers, some strategies are pointed out for the implementation of interprofessional training spaces, such as: the use of problem-based learning and online learning; flexible curriculum structure; working groups that support the proposal, as well as professors and university directors; financial support for the implementation and maintenance of interprofessional education activities; participation of students, teachers and service professionals for the evaluation and construction of the curricula for the courses^(^ 38 ^)^.

The study contributions indicate the importance of gradually inserting the IECP discussion, as it is observed that, in this PRMSF, the work developed by the residents occurs in the logic of multiprofessionality. Thus, the intentional and planned work, involving the actors of the residency with the objective of incorporating the theoretical and methodological references of the IECP in the Political Pedagogical Project, is a fundamental element to enhance interprofessionality. Considering that the residency occurs at the interface of the practice scenarios, the importance of articulating health work as a determining factor for the training process in the interprofessional logic is emphasized.

Among the limitations of the study is the data collection technique, since the use of the in-person interview or the focus group could have provided a deeper understanding of the participants’ answers. In addition, the non-inclusion of other educational institutions and graduates of other multiprofessional residency programs in health limited the scope of the findings of this research.

## Conclusion

The study showed that the work developed during the PRMSF occurs in the logic of multiprofessionality with a tendency for interprofessional work triggered by actions that involve the theoretical methodological framework of the IECP realized in the case discussion with all the professional categories of the residency, in the practical experiences that occur during a shared consultation, at home visits, in health education groups and in inter-sectoral actions.

It is worth mentioning that the aforementioned activities need to be proposed intentionally for the residency program and aligned with the practice scenarios, as it is believed that, in this way, the IECP provides the training of professionals capable of collaborative work.

In the context of the residency, interprofessionality is shown to be a relevant strategy for professional training and to improve quality of care, based on shared care planning, the user as the center of care, learning about the other professions, and the exchange of knowledge with the other.
